# Updates on Antiobesity Effect of *Garcinia* Origin (−)-HCA

**DOI:** 10.1155/2013/751658

**Published:** 2013-08-06

**Authors:** Li Oon Chuah, Wan Yong Ho, Boon Kee Beh, Swee Keong Yeap

**Affiliations:** ^1^School of Industrial Technology, University Science Malaysia, 11800 Penang, Malaysia; ^2^School of Biomedical Sciences, The University of Nottingham Malaysia Campus, Jalan Broga, 43300 Semenyih, Selangor, Malaysia; ^3^Department of Bioprocess Technology, Faculty of Biotechnology and Biomolecular Sciences, University Putra Malaysia, 43400 Serdang, Selangor, Malaysia; ^4^Institute of Bioscience, University Putra Malaysia, 43300 Serdang, Selangor, Malaysia

## Abstract

*Garcinia* is a plant under the family of Clusiaceae that is commonly used as a flavouring agent. Various phytochemicals including flavonoids and organic acid have been identified in this plant. Among all types of organic acids, hydroxycitric acid or more specifically (−)-hydroxycitric acid has been identified as a potential supplement for weight management and as antiobesity agent. Various *in vivo* studies have contributed to the understanding of the anti-obesity effects of *Garcinia*/hydroxycitric acid via regulation of serotonin level and glucose uptake. Besides, it also helps to enhance fat oxidation while reducing *de novo* lipogenesis. However, results from clinical studies showed both negative and positive antiobesity effects of *Garcinia*/hydroxycitric acid. This review was prepared to summarise the update of chemical constituents, significance of *in vivo*/clinical anti-obesity effects, and the importance of the current market potential of *Garcinia*/hydroxycitric acid.

## 1. Introduction

The world is in health transition. Infection as a major cause of suffering and death is giving way to new epidemics of noncommunicable disorders such as cancer, cardiovascular diseases, and diabetes, which continue to plague the world at an alarming rate [1]. A trend of increasing prevalence of obesity and obesity-related comorbidity and mortality was observed over the last few decades [2]. The International Association of the Study of Obesity (IASO) reported on the country rankings in terms of percentage global prevalence of adult obesity (BMI ≥ 30 kg/m^2^) in the year of 2012, where Tonga ranks first with 56.0% obese adults (46.6% of obese male and 70.3% of obese female). In the United States, IASO reported that 35.5% of men and 35.8% women were obese (BMI ≥ 30) [1, 3]. Overweight and obesity are diagnosed based on the body mass index (BMI), which is defined as quotient of body weight (kg) over the square of stature (m^2^). According to the World Health Organization (WHO) standard, overweight subjects are diagnosed with BMI values in the range of 25–29.99. Obesity itself, defined as BMI ≥ 30, is associated with several chronic and debilitating health problems including hyperlipidemia, hypertension, coronary heart disease, diabetes, cancer, disease of the gall bladder, osteoarthritis, shortage of breath, abnormal dilation of the veins, backache, and even psychological problems [2, 4]. 

There are a few drugs in the market to ameliorate or prevent obesity, but there are costs, efficacy, and side effects to be considered. For example, the currently available pharmacological agents, Sibutramine, Rimonabant, Orlistat, and Phentermine which are licensed for weight reduction therapy, appear to possess some adverse effects [5–7]. Phentermine, for instance, has been reported to cause dry mouth, insomnia, headache, dizziness, fatigue, and palpitation [6, 7]. In year 2010, FDA had announced the market withdrawal of Meridia (Sibutramine) due to its risk of serious cardiovascular events [6, 7]. Natural products and plant-based dietary supplements have been used by people for centuries. Evidence is starting to emerge to shed light on the consumption of herbs as an effective strategy for disease treatment and health maintenance. Several ethnobotanical studies have reported the bioprospecting surveys on the positive application of herbs in the treatments for obesity [8]. *Garcinia *has been used for centuries in Asian countries for culinary purposes as a condiment and flavoring agent in place of tamarind or lemon and to make meals more filling [9, 10]. Besides its use as a flavouring agent, the dried rind of *G. cambogia* combined with salt and other organic acids can help to lower the pH and thus provides a bacteriostatic effect in curing fish. *G. cambogia* contains large amounts of hydroxycitric acid (HCA). Similar to *G. cambogia*, *G. atroviridis* and *G. indica* also contain significant HCA content and are sometimes used interchangeably with *G. cambogia* in food preparation. The different features among these three different types of *Garcinia* are summarised in Table 1 [9, 11–14]. 

A myriad of health effects have been attributed to *Garcinia* (including *G. cambogia, G. atroviridis*, and *G. indica*), such as antiobesity effects [15–17], antiulcerogenic [18–20], antioxidative [21–24], antidiabetes [25], antimicrobial [22, 26–28], antifungal [29], anti-inflammatory [30, 31], and anticancer effects [22, 32–34]. In particular, the antiobesity effects of *Garcinia* or more specifically of its HCA content have been elucidated with unprecedented clarity over the last few decades. Besides its efficacy in the reduction of body weight and food intake, *Garcinia*/HCA has been proven to be beneficial in ameliorating obesity-related complications such as inflammation, oxidative stress, and insulin resistance [21]. The results obtained from several studies supported the positive effects of HCA administration alone or in combination with other ingredients on body weight loss, reduced food intake, increased fat oxidation, or energy expenditure (EE) [16, 17, 35–39] whereas some studies did not [40–42].

In spite of the vastly reported prominent role of HCA in inducing satiety, reduced energy intake and weight gain, and improved blood parameters and substrate oxidation, controversial results regarding its efficacy and safety as an antiobesity dietary supplement had also been reported. Evidence from the *in vitro*, *in vivo*, and clinical trials on the safety of *Garcinia*/HCA as a dietary supplement for treating obesity had been extensively reviewed [43]. However, the efficacy of *Garcinia*/HCA remains the subject of debate. Despite the previously stated issues, on conclusive evidence for HCA's efficacy in promoting weight loss and suppressing food intake, the marketing of a plethora of over-the-counter slimming aids containing HCA has taken place. The aim of this review is to critically assess the evidence from a very broad range of reports, rigorous clinical trials, systematic reviews, and meta-analyses on the efficacy and potential of *Garcinia*/HCA as an antiobesity dietary supplement.

## 2. Uses in Traditional Medical Systems

Botanical dietary supplements usually contain a complex mixture of phytochemicals which have additive or synergistic interactions. Aside from its use as a preservative and as a condiment in cuisine, *Garcinia *extract has been used in the traditional Ayurvedic medical system [9, 13]. A decoction of *G. cambogia* is given as purgative in the treatment of intestinal worms and other parasites, for bilious digestive conditions, for dysentery, rheumatism, and in the treatment of tumours. Less commonly, extracts are employed as cardiotonics to treat angina. In veterinary medicine, it is used as a rinse for diseases of the mouth in cattle [12, 77]. The fruit rind is used in rickets and enlargement of spleen and to heal bone fractures [13]. In Southeast Asian folkloric medicine, a decoction of *G. atroviridis* (leaves and roots) is sometimes used for the treatment of cough, dandruff, earache, stomach pains associated with pregnancy, and throat irritation [49]. The dried fruit of *G. atroviridis* is used for improving blood circulation, for the treatment of coughs, as a laxative, and as a expectorant. The fruit is used in a lotion with vinegar to rub over the abdomen of women after confinement [9]. Fruit of *G. indica* is antiscorbutic, cholagogue, cooling, antibilious, emollient, and demulcent. The anthelmintic properties of the fruit of *G. indica* contributed to its use in haemorrhoids, dysentery, tumor, pains, and heart complaints. Bilious affected sites are treated with syrup from the fruit juice. Kokum butter is astringent and demulcent and is used in diarrhea and dysentery. It is also applied externally for ulcerations, sinuses, fissures of hand, lip, chapped skin, and skin diseases [12, 13, 44, 77]. 

## 3. Phytoconstituents

**Figure 1:**
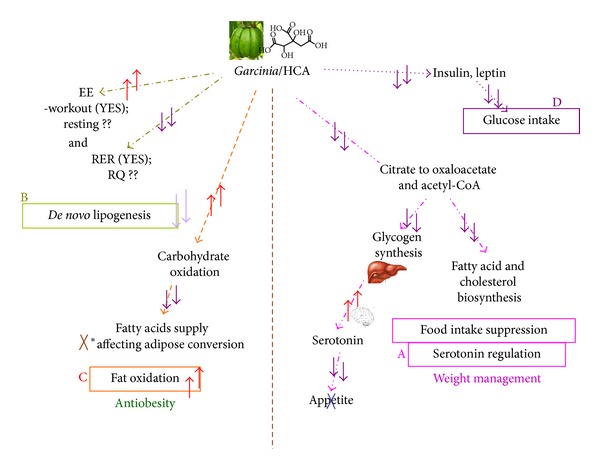
Possible multiple mechanisms that contribute to antiobesity effect of *Garcinia*/HCA. **↑** indicated increase or stimulation; **↓** indicated reduce or inhibition while ?? indicated that the effect is yet to be confirmed. (A) summary of Serotonin regulation and food intake suppression; (B) summary of reduction of *de novo* lipogenesis; (C) summary of stimulation on fat oxidation; (D) summary of reduce on glucose intake; (A) and (B) contribute to the weight management effect of *Garcinia*/HCA while (B) and (C) contribute to antiobesity of *Garcinia*/HCA.

The several compounds which have been isolated from various species of *Garcinia* are summarised in Table 2. Several types of organic acids such as HCA, citric, tartaric, malic, and succinic acids are isolated from *Garcinia*. However, HCA is the principal acid of the fruit rinds of *G. cambogia*, *G. indica, *and *G. atroviridis* [12, 27, 45], with its content ascending as listed [46–48]. A substantial amount of (−)-HCA, up to 30% by weight is present in the pericarp of the fruit of *G. cambogia*. In similar studies conducted by Sullivan et al. [78, 79] and Stallings et al. [80], they observed that of the four isomers of HCA [(–)-HCA, (+)-HCA, (–)-allo-HCA, and (+)-allo-HCA], (−)-HCA, which is also known as (2S, 3S)-HCA, was the only potent inhibitor of ATP citrate lyase. (−)-HCA can be chemically synthesized using citric acid as starting material. Synthetic (−)-HCA offers several advantages including higher purity and lactone stable as compared to natural (−)-HCA [81]. On the other hand, (−)-HCA is a good starting material to synthesize other important chiral synthons and compounds [82]. 

(−)-HCA is one of the important supplements for antiobesity and weight management. Its effect on weight management is mainly contributed by giving the feeling of full and satisfaction while the antiobesity effect is by reduction of *de novo* lipogenesis and acceleration of fat oxidation (Figure 1). In this paper, we aimed to review the mechanism for antiobesity and weight management effects by (−)-HCA (hereafter referred to as HCA)/*G. cambogia*/*G. atroviridis*/*G. indica* extracts and the assessment of these effects in the clinical settings.

## 4. Salts of HCA

On account to the discovery of (−)-HCA as an effective compound in weight management, market demand for the acid has increased tremendously. The commercially available *G. cambogia *extracts which contain approximately 50% (−)-HCA are prepared from the fruit rind [12, 69]. HCA can exist as a free acid or in the lactone form. The former form is considered to be biologically active. However, the free acid is unstable and is usually converted to its less active lactone form to attain higher stability. To prevent the cyclization of HCA into its less potent lactone, the acid has been combined with various counter ions to form stable salts [83]. 

Commercial HCA is available in free acid form and as single, double, or triple salts. Preparations with different counter ions contribute to different degree of solubility as well as bioavailability [84]. For example, Na^+^ salt of HCA had been shown to be more effective than its lactone in inhibiting lipogenesis. However, Na^+^ salt is highly hygroscopic when bound to (−)-HCA, which would deemed unfavorable for the production of pharmaceuticals for dry delivery [85]. 

To address the need to achieve higher solubility and stability, recent approaches have been focused more on the preparation of (−)-HCA in the form of a double or triple salt. Similar to its single salts, these double or triple salts also serve as good supply for essential ions [84]. A remarkable example of these would be the Ca^2+^/K^+^ salt of (−)-HCA (HCA-SX) or Super CitriMax. In contrast to the single salts, HCA-SX is completely soluble in water and thus confers higher bioavailability [84]. A number of studies on the safety of HCA-SX had been reported [43]. Daily intake of HCA-SX at this dosage was shown to be effective in reducing body weight and BMI of healthy and obese adults after clinical trials of 8 weeks [16, 64]. Gene expression studies also provided additional evidence for the safety of HCA-SX, where genes essential for mitochondrial/nuclear proteins and for fundamental support of adipose tissue were shown to be independent of the regulation by HCA-SX [17, 86]. 

A typical reduction of food appetite and an increased serotonin availability were observed in all the weight control studies of HCA-SX on both animal and human subjects. These were associated with reduced levels of total cholesterol, LDL, triglycerides, and serum leptin as well as increased HDL level and urinary excretion of fat metabolites [15, 16, 64, 84, 87]. In rats, the salt also caused downregulation of genes encoding abdominal fat leptin while expressions of the plasma leptin genes remained unaltered [17]. Nevertheless, it was postulated that a set of obesity regulatory genes [84] and inhibition to the uptake of [^3^H]-5-HT release in the brain [15] are involved in the appetite suppressing activity of HCA-SX. 

In relation to this, gene expression profiling carried out by a research group demonstrated the modulation of a specific set of genes (about 1% of 9960 genes and ESTs) in the adipocytes by dietary HCA-SX supplementation [17]. Further study on cultured mature human adipocytes revealed significant upregulation of 366 and downregulation of 348 the fat- and obesity-related genes [88]. Notably also in the microarray analyses, HCA-SX demonstrated a distinct effect on appetite suppression whereby genes encoding serotonin receptors were shown to be selectively upregulated by the salt [17]. Besides, HCA-SX was also found to be capable of activating hypoxia inducible factor (HIF), a transcription factor involved in energy metabolism [88] and restored the increase in oxidative stress, inflammation, and insulin resistance in obese Zucker rats [21]. 

## 5. Antiobesity Effects of *Garcinia*/HCA

Obesity, particularly caused by accumulation of visceral fat, is a serious risk factor of various life-style diseases such as coronary heart disease, diabetes, hyperlipidemia, hypertension, and cancer [2, 4]. Human obesity is influenced by genetic and environmental factors and particularly by changes in diet and physical activity, which contributes greatly to the development of insulin resistance, a most common underlying abnormality in human obesity [89]. Studies on food sources exerting antiobesity effects have focused on the development of herbal extracts or functional food which can suppress the accumulation of body fat. Several studies were conducted to provide scientific basis on the extensive usage of *G. cambogia* and *G. atroviridis* associated with high-fat diet- (HFD-) induced obesity where dyslipidemia, fatty liver, insulin resistance, and hyperleptinemia were acquired along with the overexpression of leptin, TNF-*α*, resistin, PPAR*γ*2, C/EBP*α*, and SREBP1c genes in epididymal adipose tissue. The effect of *G. cambogia* was largely attributed to its HCA content [90, 91]. Subsequent researches proved that the antiobesity effects of *G. cambogia*/HCA resulted from the combined actions of several mechanisms including suppressing *de novo* fatty acid biosynthesis and appetite [16, 60] and increasing energy expenditure [39], subsequently reducing body fat accumulation and weight gain in experimental animals [37, 38, 92]. In this review, we arranged the antiobesity effects of *Garcinia*/HCA based on their distinct mechanisms: (1) serotonin regulation and food intake suppression, (2) decreased *de novo* lipogenesis, (3) increased fat oxidation, and (4) downregulation of a spectrum of obesity-associated genes. 

### 5.1. Serotonin (5-Hydroxytryptamine, 5-HT) Regulation and Food Intake Suppression

HCA, the primary acid in the fruit rinds of *G. cambogia*, *G. atroviridis*, and *G. indica* [45], has been reported as the active ingredient in inhibiting ATP citrate lyase (EC 4.1.3.8) [78, 93]. ATP citrate lyase, which is an extramitochondrial enzyme catalyzing the cleavage of citrate to oxaloacetate and acetyl-CoA, was inhibited by HCA. Thus, the availability of two-carbon units required for the initial steps of fatty acid and cholesterol biosynthesis during carbohydrate feeding was limited [78, 79, 94–97]. As a result, the consumed carbon source was diverted to glycogen synthesis in liver. A signal was then sent to the brain due to this metabolic alteration, resulting in rising of serotonin level concomitant with a reduced appetite. HCA might exhibite its anorectic effect by a second possible mechanism, namely, reducing acetyl CoA, subsequently decreasing malonyl CoA levels and thereby reducing negative feedback on carnitine acyltransferase (CPT-1). The substrate of CPT-1, long-chain acyl CoA(s), may act as a mediator(s) of appetite [98, 99]. More recently, neuropeptide Y (NPY) had also been implicated in the appetite suppression of HCA. Basal concentration of the neurotransmitter was claimed to be significantly reduced in the hypothalamic tissues as a result of supplementation with HCA-SX [84]. However, the role of NPY in this is still vague to date. Several reports supported the serotonin regulation of HCA. Ohia et al. [100] demonstrated that HCA-SX enhanced serotonin availability in isolated rat brain cortex by acting as a mild serotonin receptor reuptake inhibitor (SRRI), without stimulating the central nervous system. Kaur and Kulkarni [36] conducted a study to elucidate the effect of OB-200G, a polyherbal preparation containing aqueous extracts of *G. cambogia*, *Gymnema sylvestre*, *Zingiber officinale*, *Piper longum, *and resin from *Commiphora mukul *on the modulation of food intake by serotonin modulators in female mice. The results obtained were compared with fluoxetine, a drug that was reported to enhance 5-HT neurotransmission [101]. Both OB-200G and fluoxetine significantly (*P* < 0.05) antagonized the hyperphagic effect of p-chlorophenylalanine (PCPA), 8-hydroxy-2-(di-N-propylamino)-Tetralin (8-OH-DPAT), cyproheptadine, and 2-deoxy-D-glucose (2-DG) which further instigate possible serotonergic involvement in the effects of OB-200G on food intake in female mice. Preuss et al. [16] reported that HCA caused a significant reduction in appetite, weight loss, and plasma leptin level, concomitant with an increase in the serum serotonin level and a favorable lipid profile in human clinical trials. Similar results were also obtained in a study conducted by Asghar et al. [87] They reported on increased brain serotonin level in obese Zucker rats receiving *G. cambogia* extract, suggesting that the ability of HCA in body weight gain reduction was most probably due to its combined effects on the metabolic and serotonin pathways. In addition, Roy et al. [17] reported that HCA-SX supplementation upregulated prostaglandin D synthase (PDS), aldolase B (AldB), and lipocalin (LCN2) genes in abdominal fat tissue. Further mapping of the candidate genes of known pathways associated with fat metabolism by using functional categorization and pathway construction software showed that supplementation of HCA-SX targeted on the serotonin receptor. 

Leonhardt et al. [102] reported that HCA reduced body weight regain in rats after a period of substantial body weight loss. Besides, HCA temporarily reduced food intake of rats with diets of varying nutrient contents (grounded standard rat chow, high glucose, and high glucose + fat). HCA supplementation caused pronounce suppression of food intake during the entire 10 days of ad libitum feeding period in rats fed with high glucose + fat diet, a diet that had a nutritionally relevant level of dietary fat (24% of energy). These data therefore extended those of the previous studies which reported on the anorectic effects of HCA [96, 97, 103–105]. Moreover, the results obtained were consistent with studies which reported on particularly strong food intake suppression by HCA with high glucose + fat diet and a smaller but still significant suppression with the high glucose diet in other rat models and in different orders [37, 39, 102]. Hence, the feed conversion efficiency [cumulative body weight regain (g)/cumulative food intake (MJ)] in the high glucose and high glucose + fat groups during the 10 ad libitum days was reduced, which indirectly supported that HCA increased energy expenditure in these groups. 

Leonhardt and Langhans [39] then extended their study on the long-term effects of HCA on body weight regain and food intake, as well as the effects of HCA on the circadian distribution of food intake and on meal patterns during the dark and light phases. HCA administration significantly reduced the food intake of rats fed with 12% fat diet, but not 1% fat diet, concomitant with significant reduction in weight regain (overall *P* < 0.01) in both groups. In the study, the rats underwent restrictive feeding for 10 days prior to ad libitum feeding (Experiment 1: normal 1% fat diet and 1% fat diet + 3% HCA; Experiment 2: normal 12% fat diet and 12% fat diet + 3% HCA). The control groups of both experiments had compensated the body weight loss, whereas the HCA-fed rats groups regained only 68 ± 4% (1% fat diet) and 61 ± 8% (12% fat diet) of the body weight regained by their respective control groups after 22 days of such ad libitum feeding. Despite significant reduction in weight regain in rats fed with 1% and 12% fat diet, long-term suppression of HCA on food intake was only detected in combination with 12% fat diet (Experiment 2). This was in line with the results obtained by Leonhardt et al. [106] who suggested that HCA increased energy expenditure. Studies on the effects of HCA on the circadian distribution of food intake and on meal patterns showed that the suppression of food intake occurred predominantly during the dark phase of the first ad libitum days. However later on, HCA suppression of food intake was more effective during the light phase. Further experiments elucidating the effects of HCA in combination with the 12% fat diet on meal size and meal number during the light phase revealed that HCA markedly reduced the meal number, but not the meal size. HCA did not affect any metabolic variables tested (plasma glucose, lactate, triacylglycerol, HDL, free fatty acids, *β*-hydroxybutyrate, and insulin, hepatic fact, and glycogen concentrations) in both experiments, except decreasing plasma triacylglycerol levels and increasing the liver fat concentration in Experiment 2 (rats fed with 12% fat diet). The fact that HCA did not affect plasma *β*-hydroxybutyrate (BHB) levels did not support the hypothesis that HCA suppressed food intake via increased hepatic fatty acid oxidation. 

However, contradicting results were obtained by Kovacs et al. [41, 71] who reported that two-week supplementation with HCA and HCA combined with medium-chain triglycerides did not result in increased satiety. The findings were in line with previous reports where no significant treatment effects were observed on appetite indices (inclusive of mean, peak or nadir hunger ratings, mean ratings of desire to eat, prospective consumption, fullness or sensations of thirst, stomach growling, headache, distraction, irritability, or, as a check on malingering, itchiness) [69]. The lack of efficacy and transient food intake suppression by HCA raised questions about its clinical significance. While negative findings are always open to methodological questions, several questions need to be answered before drawing a definite conclusion. First, the diet administered to the subjects should not promote extreme sensations in the evaluation of the food intake suppression effects of HCA under conditions of energy restriction. However, Mattes and Bormann imposed mild restrictions and thus ruled out this possibility as evidenced by ratings falling in the middle range of the response scales. Second, an energy-restricted diet would prevent the required enzyme alterations (reduction of acetyl-CoA and suppression of formation of carnitine palmitoyltransferase I inhibitor malonyl CoA) which altered substrate metabolism and satiety. However, it was unlikely that the moderate energy restricted diet prescribed in the study conducted by Mattes and Bormann [69] hindered the satiety effect of HCA as it still contained at least 30% of energy from fat. 

Several factors might contribute to the controversial results of the efficacy of HCA in human studies. One of contributing factors is the highly variable doses used in the human trials which ranged from 5 to 250 mg/kg of HCA per day [42, 70]. Besides, the discrepancy might also be due to the differences in the preparation or extraction of HCA. For instance, the extraction method might increase the formation of HCA in a lactone form, which is less potent in the inhibition of ATP citrate lyase [85, 107]. In order to prevent the cyclization of HCA into the less potent lactone form, preparation using different counte rions (such as potassium, sodium, or calcium) had been applied [84], which contributed to the different degrees of stability, bioavailability, or solubility of HCA [86]. In this respect, Louter-Van De Haar et al. [83] conducted a study on the efficacy of three commercially available HCA products on suppression of food intake in male Wistar rats. Many human studies which reported lack of efficacy used Super CitriMax at considerably lower doses [41, 70, 71]. On the contrary, Preuss et al. [16] reported that high doses of Super CitriMax exerted significant effects in human. Thus, Louter-Van De Haar et al. [83] suggested that the reported lack of efficacy of HCA in suppressing food intake in human subjects might be due to the low doses of a relatively low-effective HCA preparation. Nevertheless, significant suppression of food intake was observed in the studies conducted by Leonhardt and Langhans [39] where Sprague-Dawley rats were supplemented with HCA for 10 days after substantial, fasting-induced weight loss. It seemed that HCA might be more effective in regulating weight gain than promoting weight loss; thus it was more useful for weight maintenance after an initial loss [39, 102]. Hence, differences in the experimental setups such as the difference in rat strains could contribute to such discrepancy. 

### 5.2. Decreased *De Novo* Lipogenesis

The reduction of the acetyl-CoA by HCA and thus limiting the availability of building blocks required for fatty acid and cholesterol biosynthesis has led to suggestions that HCA inhibited lipogenesis. Several studies conducted by Sullivan and colleagues had confirmed the inhibition of *in vivo* and *in vitro* rates of lipogenesis in several tissues reported to convert carbohydrate into fatty acids (such as liver, adipose tissue, and small intestine), in which HCA was predominantly given to rodent models [78, 79, 85, 96, 97, 108]. Lowenstein [108] demonstrated that HCA greatly inhibited *in vivo* fatty acid synthesis in rat liver. The rats were placed on chow diet for 7–10 days, followed by 45 h of fasting prior to a scheduled diet high in fructose or glucose for 10 to 15 days. The sodium salt of HCA at dose levels of 2 to 20 mM was administered by intraperitoneal injections 45 min before injection of ^3^H_2_O. Fatty acid biosynthesis in rat liver (*μ* moles ^3^H_2_O incorporated/g liver/h) was measured 3.5–5 h after starting of the final feeding. Profound decrease in fatty acid synthesis by 25 to 30 days was obtained with an intraperitoneal dose of 0.1 mmole per kg of body weight (equivalent to approximately 2.9 mg of HCA per 150 g rat). In addition, 50% of inhibition was detected at a dose level of 0.28 mmole per kg body weight. 

It was reported that *G. cambogia*/HCA affected respiratory quotient (RQ) and EE in rats and human. Lim et al. [109, 110] showed that short-term administration of HCA decreased the RQ in athletes and in untrained women. Leonhardt et al. [106] further extended their study to determine the effect of HCA on RQ and EE in rats fed ad libitum after a period of substantial weight loss. They reported that HCA markedly decreased RQ and EE during the first two days of ad libitum, reflecting suppression of *de novo* lipogenesis in rats, which is consistent with the findings of Westerterp-Plantenga and Kovacs [61] in humans. 

In this respect, Kovacs and Westerterp-Plantenga [60] further extended their study where the effects of HCA on net fat synthesis as *de novo* lipogenesis were investigated. A double-blind, placebo-controlled, randomized, and crossover design experiment was conducted on 10 sedentary male subjects. The subjects performed glycogen depletion exercise, followed by a 3-day high-fat low-carbohydrate (F/CHO/P, 60/25/15% energy; 100% of EE; depletion period) intake in order to create a similar glycogen storage capacity. Subsequently, a 7-day high-carbohydrate diet (F/CHO/P, <5/>85/10% energy; 130–175% of EE; overfeeding period) supplemented with either 500 mg of regulator HCA (HOB Ireland Ltd.) or placebo was administered. Each intervention ended with a 60 h stay in the respiratory chamber (days 9 and 10). *De novo* lipogenesis occurred as indicated by RQ > 1.00 in all subjects. Significantly, lower 24 h EE (*P* < 0.05; on day 9), resting metabolic rate (*P* < 0.01; on day 10), and RQ at night (*P* < 0.05; on day 10) were detected with HCA as compared to placebo. Fat balance and thus net fat synthesis as *de novo* lipogenesis tended to be lower (*P* < 0.1) with HCA as compared to placebo. Taken all together, Kovacs and Westerterp-Plantenga concluded that the administration of HCA during overfeeding of carbohydrates may reduce *de novo* lipogenesis. 

However, opinions differ widely with respect to this issue. The mechanism underlying the anorectic effect of HCA is still unclear. Furthermore, whether the suppression of body weight regained was solely due to reduced food intake or whether there was involvement of increased EE remained unknown. Contradictory results were reported on the effects of HCA on EE. Previous reports by Leonhardt and colleagues [39, 102] and the results obtained in pair-feeding studies [103] showed reduction of body weight regain and energy conversion ratio by HCA supporting the finding that HCA increased EE. However, reduced energy conversion ratio could be due to decreased nutrient absorption. Vasselli et al. [111] demonstrated an increment in 24 h EE in rats fed with mixed high-carbohydrate diet ad libitum by directly measuring the EE in a whole-body respirometer, albeit no effect on the RQ was detected. Another study conducted by Leray et al. [112] reported that 6 months of HCA administration did not affected EE in adult neutered cats. Besides, most human studies [41, 42, 70] reported that HCA had no effect on EE. Kriketos et al. [42] reported that HCA administration exhibited no effect on lipid oxidation in men during either rest or moderately intense exercise on a cycle ergometer. However, in these studies, the subjects received a much smaller dose, namely, a daily dose of 3.0 g per subject [nearly equal to 1.5 mg/day/mouse]. Furthermore, their experimental period of 3 days was quite short when compared with other studies. 

Blunden [113] reported that when *Garcinia* extract and insulin were added simultaneously, the number of larger droplets markedly decreased while the smaller droplets (10–20 *μ*m^2^ or <10 *μ*m^2^) increased in 3T3-L1 cell. The activity of cytosolic glycerophosphate dehydrogenase (GPDH) which converts dihydroxyacetne phosphate to glycerol 3-phosphate (predominant substrate for triglyceride synthesis) increased from undetectable levels to between 100 and 187 U/mg of cytosolic protein after adipose conversion. However, no significant decrease in enzymatic activity was detected after administration of the *Garcinia* extract. Taken together, the authors therefore suggested that *Garcinia* extract interferes with lipid synthesis in fat cells via fatty acid supply inhibition without affecting adipose conversion. 

### 5.3. Increased Fat Oxidation

Ishihara et al. [35] conducted a study on acute and chronic effects of HCA on energy metabolism. Acute administration of 10 mg/100 *μ*L of a 0.48 mol/L HCA solution per mice significantly increased (*P* < 0.05) serum free fatty acid levels and concentration of glycogen in the gastrocnemius muscle, even though the respiratory exchange ratio was not different from that in the control group. On the other hand, chronic administration of 10 mg HCA twice a day significantly lowered (*P* < 0.01) the RQ during resting and exercising conditions in mice. Lipid oxidation, calculated from RQ, and oxygen consumption were significantly enhanced, and carbohydrate oxidation was significantly less in these mice during the early stages of running (*P* < 0.01). Taken all together, the authors therefore suggested that chronic administration of HCA augmented the endurance exercise performance in mice via the attenuation of glycogen consumption caused by the promotion of lipid oxidation during running exercise. Furthermore, Ishihara et al. [35] suggested that chronic HCA administration might have increased EE during the 3-week experimental period. 

In addition, Lim et al. [109, 110] also showed that short-term administration of HCA increased fat oxidation during exercise in athletes and in untrained women. Lim et al. [110] conducted a randomized, placebo-controlled study where subjects (athletes) consumed HCA (250 mg) or placebo for 5 days, after each time performing cycle ergometer exercise at 60% VO_2_ max for 60 min followed by 80% VO_2_ max until exhaustion. The results obtained showed that the respiratory exchange ratio (RER) was significantly lower in the HCA trial than in the control trial (*P* < 0.05). Fat oxidation was significantly increased by short-term administration of HCA, and carbohydrate oxidation was significantly decreased (*P* < 0.05) during exercise in athletes. In a continuation of their study, Lim et al. [109] conducted a similar study to evaluate the effects of HCA administration on fat oxidation during exercise in untrained women. The results showed that HCA decreased the RER and carbohydrate oxidation during 1 hour of exercise. In addition, exercise time to exhaustion was significantly enhanced (*P* < 0.05). 

A more recent approach for determining fat metabolism by HCA was conducted by measuring urinary concentration of malondialdehyde (MDA), acetaldehyde (ACT), formaldehyde (FA), and acetone (ACON) of the tested subjects. The urinary excretion of these four metabolites was proposed to be a consequence of enhanced *β*-oxidation of fats in body tissues [16]. The effect of HCA-SX had been studied extensively by Preuss et al. on obese human subjects [16, 64] as well as on male and female Sprague-Dawley rats. In the randomized, double-blind, and placebo-controlled clinical studies on obese human, a group of subjects were given 4,667 mg of HCA-SX daily (provided 2,800 mg HCA/day) while the other given a combination of HCA-SX 4,667 mg, 4 mg of niacin-bound chromium (NBC), and 400 mg of gymnema sylvestre extract (GSE) daily. The control group received placebo in 3 equal doses daily at 30 to 60 min before meals. In the trial involving 30 subjects, urinary excretion of fat metabolites was increased by approximately 125–258% whereas in trial involving 60 and 90 obese subjects, the metabolite excretion increased by about 35.6–106.4% [16] and 32–109% [64], respectively. As excretion of fat metabolites was enhanced in groups receiving the combination formula, it was also suggested that HCS-SX, either alone or in combination with NBC and GSE, could effectively promote breakdown of fats [16, 64]. 

### 5.4. Downregulation of a Spectrum of Obesity-Associated Genes

Lipogenic transcription factors, including SREBP1c, liver X receptors, PPAR*γ*, and C/EBP*α*, are highly expressed in the adipose tissue and actively participate in the lipid metabolism of adipocytes by coordinating lipogenic and adipocyte-specific gene expression [114]. PPAR*γ* interacts with several other transcription factors. C/EBP*α* and PPAR*γ* interact via a positive feedback loop in the differentiated adipocytes, to induce each other's expression [115]. Besides, coexpression of PPAR*γ* with SREBP1c increases the transcriptional activity of PPAR*γ* [116]. aP2 (a marker of terminal adipocyte differentiation), together with several adipocyte-specific genes, including adiponectin, insulin receptor, leptin, glucose transporter 4 (GLUT4), and glycerol phosphate dehydrogenase, are induced during the adipogenic differentiation process [114]. Leptin, a 167-amino acid hormone and a biomarker of the obesity regulatory gene, is produced by fat tissue and is known to regulate energy intake and metabolism. Leptin binds to the medial nucleus of the hypothalamus and induces a sensation of satiety and thus controlling the appetite [98, 117, 118]. 

Fatty acid synthase, acetyl-CoA carboxylase 1, and SREBP1c mRNA concentrations were decreased in the adipose tissue of the obese animal models [119]. On the contrary, the mRNA and protein expression of TNF*α* (which is involved in proinflammation, apoptosis, lipid metabolism, and insulin resistance) were increased in the adipose tissues of the obese rodents and humans [120]. A high level of TNF*α* suppressed transcription factors such as PPAR*γ* and C/EBP*α* which, in turn, activated the GLUT4 gene [121, 122].

Hayamizu et al. [123] evaluated the effects of *G. cambogia* fruit rind extract containing 60% (–)-HCA on serum leptin and insulin in mice. *G. cambogia* extract reduced serum total cholesterol, triacylglycerol, and nonesterified fatty acids in mice. Nevertheless, the body weight gain and fat pad weight were not affected in the treatment. No significant difference in blood glucose level was detected between groups, but a significant reduction of serum insulin (*P* < 0.05) was detected, suggesting that the *G. cambogia* extract efficiently improved glucose metabolism in the treated animals. In addition, the treatment decreased serum leptin levels and the leptin/WAT ratio. Besides, the changed ratio of body weight correlated positively with leptin levels in their study. Furthermore, it had been reported that leptin suppressed the signal transduction of insulin via cytokine interactions [124, 125]. Hayamizu et al. [123] suggested that the observed effect of *G. cambogia* extract on serum insulin in their study occurred through leptin-like activity. 

The antiobesity effects of *Garcinia* on visceral fat mass, lipid profiles in the serum and liver, serum adipocytokine levels, and regulation of the expression of multiple adipose tissue genes were reviewed. Kim et al. [37] reported the antiobesity effects of a mixture composed of aqueous extract of *G. cambogia*, soy peptide, and L-carnitine (1.2 : 0.3 : 0.02, w/w/w) on rats rendered obese by high-fat diet (HFD). An HFD (40% fat calories) with identical composition of the high-fat control diet (CD) applied in the study was fed to five-week-old male Sprague-Dawley rats for 9 weeks to create an obese conditions in rats that mimic to human obesity. Body weight gain, visceral fat-pad weight, and serum and hepatic biochemistry of rats were measured. The 0.38% mixture-supplemented HFD (D + M) reduced the total body weight and the accumulation of visceral fat mass and lowered the blood and hepatic lipid levels, which led to the improvement of insulin resistance in the HFD-induced obese rats. Moreover, the mixture of *G. cambogia*, soy peptide, and L-carnitine improved dyslipidemia in rat models with HFD-induced obesity. Downregulation of the expression of leptin, tumor necrosis factor-alpha, and sterol regulatory element binding protein 1c genes in the epididymal fat tissue of rats fed with CD + M diet was obtained. In contrary, upregulation of the uncoupling protein 2 (UCP2) gene in epididymal adipose tissues was induced with CD + M diet. No effect on the food intake of the animals was observed in the study, suggesting that the mixture exerted antiobesity effect via modulation of the metabolic derangement induced by HFD during which interactions between the multiple genes implicated in the process of adipogenesis might be involved, rather than simply suppressing appetite. A similar observation was obtained by Kim et al. [38], where in addition to the reduction of food intake, the food efficiency ratio (FER) was also significantly lower in the *G. cambogia* diet administrated group than in the HFD mice, implying less efficient transformation of the feed mass into body mass. 

## 6. Human Clinical Trials

The antiobesity effects of *G. cambogia* in terms of promoting weight loss and lowering cholesterol level were extensively studied. However, evidence for the effectiveness of *G. cambogia* or its derivative products was largely derived from animal studies [126]. Despite the intriguing evidence of antiobesity effects of *G. cambogia* from *in vitro* and animal studies, more equivocal results were obtained from randomized double-blind placebo-controlled experiments dealing with human subjects [127–130]. Hayamizu et al. [59] conducted a crossover design randomized controlled trials (RCTs) to determine the “no observed adverse effect level (NOAEL)” of *G. cambogia* extract in 44 healthy volunteers (22 males and 22 females) and concluded that *G. cambogia* is generally safe to be consumed. Several equivocal findings of RCTs were reported on the effectiveness of supplements containing HCA (Table 3). Some studies reported that HCA exerted no significant effects as compared to the placebo group [40, 42, 70] (Table 4). All the above findings were in agreement with the most recent meta-analysis of RCTs which revealed that *G. cambogia* extract possessed limited or no effects on weight-loss in human subjects [131]. Moreover, this study showed no effect on satiety or calorie intake in overweight individuals consuming their habitual diet, which is in agreement with past studies [41, 60, 75]. However, such comparisons must be made with caution as the variations in the formulations, doses administered, RCTs designs, and study populations might contribute to the discrepancy of the results.

Preclinical studies using rodent models have confirmed the body weight reduction, appetite suppression, and subsequently food intake reduction effects of HCA in rats. Clinically, however, HCA failed to perform well. Several factors that might contribute to this scenario are the ATP citrate lyase which might be important only at very high carbohydrate diets, a type of diet that most studies did not prescribe. Besides, a high-fiber diet can bind to HCA and block it, thus reducing its efficacy. HCA and *G. cambogia* exerted potential effects in weight management, but clinical studies have yet to prove optimum conditions for HCA to be effective. For instance, Sullivan et al. [85] reported that hepatic lipid synthesis was reduced only if HCA was administered before the beginning of feeding, reaching optimum 30–60 minutes prior to feeding. The reason for this remains unknown. 

## 7. Patents and Commercialization

The claims on enhanced human health associated with *Garcinia*/HCA had been reviewed in Section 4. In particular, the antiobesity effects of *Garcinia*/HCA were extensively reported. This has resulted in the availability of numerous commercialized weight-management products derived from *Garcinia*/HCA (Table 5). Several products of *G. cambogia* or its derivatives had been patented and commercialized. As of August 2012, a total of 66 patents that apply to *G. cambogia *or HCA derived from *Garcinia* were filed with the US Patent and Trademark Office (USPTO) since 1976 (search of US Patents and Trademark Office in year 2012 using Google patent search). These patents are on various aspects, including HCA enrichment from *Garcinia* rind, HCA and food products/dietary supplements prepared therefrom, methods of production, and their use. The majority of the patents are related to *G. cambogia/atroviridis* and/or HCA derived from *Garcinia* on obesity and weight loss. The patent numbers are as follows: 8,197,867, 8,097,286, 8,017,147, 8,003,138, 7,943,186, 7,943,183, 7,927,636, 7,858,128, 7,846,970, 7,772,428, 7,741,370, 7,687,082, 7,550,161, 7,507,421, 7,431,951, 7,335,651, 7,311,929, 7,230,131, 7,214,823, 7,208,615, 7,189,416, 7,179,488, 7,063,861, 6,982,098, 6,899,891, 6,875,891, 6,855,358, 6,770,782, 6,706,899, 6,676,977, 6,610,277, 6,565,847, 6,541,026, 6,489,492, 6,485,710, 6,476,071, 6,447,807, 6,428,806, 6,426,077, 6,413,545, 6,399,089, 6,395,296, 6,277,396, 6,221,901, 6,217,898, 6,160,172, 6,147,228, 6,113,949, 6,054,128, 5,972,357, 5,911,992, 5,783,603, 5,656,314, 5,612,039, 5,536,516, 4,007,208, 4,006,166, 4,005,086, 7,119,110, 3,994,927, 3,993,668, 7,153,877, 3,993,667, 4,028,397, 7,153,528, 7,015,250, 6,638,542, 6,579,866, 6,482,858, 6,441,041, 6,383,482, 6,207,714, 5,817,329, 5,626,849, and 3,965,121.

## 8. Conclusions

The nutraceutical industry is flourishing, and interest in establishing scientific credibility has attained importance for many companies and scientists. In the recent years, more clinical trials had been conducted to elucidate the functional effects of *Garcinia*/HCA supplementation on promoting human health. A multitude of metabolic functions had been reported for HCA or HCA-containing *Garcinia* extract, such as reducing blood lipids, inducing weight loss, suppressing appetite, and reducing food intake based on results obtained in both animal trials and human clinical trials (Figure 1). These discoveries make the development of evidence-based adjuvant modalities to curb the trend of the increasing prevalence of obesity and obesity-related comorbidity and mortality possible. We have previously reviewed and concluded that *Garcinia *extract and HCA were generally safe to be consumed. Collective results from 17 clinical studies which involved 873 subjects demonstrated the safety of HCA and HCA-SX for human consumption [43]. These studies provided scientific evidence that intake of HCA and HCA-SX alone did not produce adverse effects and a dietary dosage of up to 2800 mg/day was regarded as the “no observed adverse effect level (NOAEL)” of HCA-SX in human [84]. Based on these animal and human safety data, HCA-SX also received self-affirmed GRAS status in the USA by the Burdock Group in year 2003 [132]. However, definitive conclusions that *Garcinia*/HCA supplements are efficient tools against various health problems especially obesity remain to be proven in larger-scale and longer-term clinical trials, despite substantial public interest in such supplements. Many diet supplements containing *Garcinia*/HCA marketed as weight management products are the combination of active ingredients rather than containing a single agent. Thus it is difficult to evaluate the effectiveness of single agents when the combination products are tested. In addition, awareness of the safety and efficacy of the weight management supplements available in the market should be raised among health care providers in order to assist their patients in analyzing the risks and benefits of the dietary supplements. Thus, scientific investigations on the potential health promoting effects of herbal preparations as diet supplement are prerequisites for new discoveries of alternative therapies.

## Figures and Tables

**Table 1:** Comparison of *G. cambogia*, *G. atroviridis*, and *G. indica* [9, 11, 13, 44].

Species	Common name	Origin	Feature
*G. cambogia *	Asam Gelugor	India: found commonly in the evergreen forests of Western Ghats, from Konkan southward to Travancore, and in the Shola forests of Nilgiri.	Small- or medium-sized tree with a rounded crown and horizontal or drooping branches, under the family of Guttiferae. Its fruits are ovoid, about 5 cm in diameter, yellow or red when ripe with six to eight grooves, enclosing six to eight seeds, and are edible.

*G. atroviridis *	Asam Gelugor	Southeast Asia	Small- or medium-sized fruit tree, with drooping branches and ovoid fruits. The fruits are bright orange-yellow when ripe, globose with 12–16 grooves, about 7–10 cm in diameter, and fluted with a firmly textured outer rind and a rather thin and translucent pulp surrounding the seeds.

*G. indica *	Kokum	India: the tropical rain forests of Western Ghats, from Konkan southward to Mysore, Coorg, and Wayanad	Slender evergreen tree with drooping branches. Its fruits are globose or spherical, 2–4 cm in diameter, dark purple when ripe with five to eight large seeds surrounded.

**Table 2:** Phytochemicals of *Garcinia. *

Phytochemicals	*G. cambogia *	*G. indica *	*G. atroviridis *
Organic acids			
(−)-HCA	Fruit rind [45, 46]	Fruit rind [45]	Fruit rind [45]
Citric acid	Fruit rind [46]	Leave and fruit rind [47]	Herbal products [48]
Tartaric acid	[46]^nd^	[47]^nd^	Herbal products [48]
Malic acid	Fruit rind [46]	[47]^nd^	Herbal products [48]
Succinic acid	—	[47]^nd^	—
Prenylated benzoquinone			
Atrovirinone	—	—	Root [27]
Prenylated depsidone			
Atrovirisidone	—	—	Root [27]
Atrovirisidone B	—	—	Root [49]
Prenylated hydroquinone			
4-Methylhydroatrovirinone	—	—	Root [50]
1,4-cis-Docosenoic acid	—	—	Root [50]
14-cis-Docosenoic acid	—	—	Root [50]
Flavonoid			
Morelloflavone	—	—	Root [50]
Fukugiside	—	—	Root [50]
Naringenin			Root [49]
3,8′′-Binaringenin			Root [49]
Xanthone			
Garbogiol	Root [51]	—	—
Rheediaxanthone A	Bark [51]	—	—
Dioxygenated xanthone			
1,7-Dihydroxyxanthone	—	Heartwood [52]	—
Tetraoxygenated xanthone			
Atroviridin	—	—	Stem bark [53]
Tetracyclic xanthone			
Oxyguttiferone K	Fruit [54]	—	—
Polyisoprenylated benzophenone			
Garcinol/camboginol (enantiomer of xanthochymol)	Fruit rinds [55, 56] Latex [57] Bark [51]	[55]^nd^ Fruit rinds [58]	—
Isogarcinol/cambogin (enantiomer of isoxanthohumol)	Latex [57] Bark [51]	Fruit rinds [58]	—
Isoxanthohumol	Fruit rinds [55]	[55]^nd^	—
Guttiferone I	Fruit [54]		—
Guttiferone J	Fruit [54]		—
Guttiferone K	Fruit [54]		—
Guttiferone M	Fruit [54]		—
Guttiferone N	Fruit [54]		—

nd: none detected; —: not reported.

**Table 3:** Summary of clinical studies of *Garcinia*/HCA that have shown significant antiobesity effect.

Duration	Subject	Treatment	Outcome	References
10 days	44 healthy volunteers	4 g for the 1st day followed by 3 g until day 10.	Not recorded	[59]

11 days	Normal, groups: placebo, HCA of total 10 sedentary male under high-fat diet	1500 mg HCA daily	No significant effects on body weight gain, appetite-related, and plasma parameters but decreased fat deposition **Comments: suggest that (−)-HCA may reduce net fat deposition from *de novo* lipogenesis during weight gain	[60]

2 weeks	BMI 27.5, groups: placebo, HCA of 24 subjects each (12 males, 12 females)	300 mg HCA	Reduced of energy intake and slight decreased of body weight	[61]

4 weeks	Obese, hypocaloric diet, groups: placebo, 1 capsule, 2 capsule, (50 subjects each group)	55 mg *G. cambogia *+ 19 mg chromium + 240 mg chitosan/day	Weight loss associated with lower TC/LDL and higher HDL	[62]

8 weeks	BMI 25–35, groups: placebo, treatment of 20 each	1000 mg HCA/day	Reduced of visceral and subcutaneous fat area	[63]

8 weeks	Moderate obese, groups: placebo, HCA-SX, HCA-SX + NBC + GSE of total 60	HCA-SX 4667 mg (60% HCA), 4667 mg HCA-SX + 400 *μ*g niacin-bound chromium + 400 mg Gymnema sylvestre extract	Significant (*P* < 0.05) decrease in BMI, food intake, total cholesterol, low-density lipoproteins, triglycerides, and serum leptin levels, increase in high-density lipoprotein levels, and enhanced excretion of urinary fat metabolites (biomarker of fat oxidation, including malondialdehyde, acetaldehyde, formaldehyde, and acetone) in HCA and HCA-NBC-GSE groups	[16]

8 weeks	BMI 30–50, groups: placebo, 2800 mg HCA; 2800 mg HCA + 400 *μ*g chromium of 30 subjects each	2800 mg HCA; 2800 mg of HCA-SX + 400 *μ*g chromium + 100 mg gymnemic acid/day	Decreased of body weight, BMI, LDL, and TG and increased fat oxidation	[64]

8 weeks	Normal, group: 35 healthy subjects on diets 1000, 1200, or 1500 kcal	1500 mg *G. cambogia *extract daily	Significant reduction of total cholesterol, triacylglycerol, and body weight associated with reduced appetite	[65]

8 weeks	Obese, groups: placebo and treatment of total 50 F	3.45 g *G. atroviridis *	Significant reduction of body weight, BMI, body fat, lean body mass, and anthropometric parameters (biceps, subscapular, suprailiac crest skinfold thicknesses, and upper arm circumference) but no change of serum lipid profile	[66]

60 days	Over weight to obese, groups: placebo and treatment of total 58	285 mg (−)-HCA in Slim339 *G. cambogia* extract daily	Significant reduced of body weight (4.7%)	[67]

12 weeks	BMI 27.5–39, groups: placebo and treatment (18 females/2 males each) of total 40	300 mg *G. cambogia* + 1200 mg *Phaseolus vulgaris *+ 1200 mg inulin/day	Significant body weight lost (3.5 kg versus 1.2 kg)	[68]

12 weeks	BMI ~28, groups: placebo: 47; treatment: 42	2400 mg *G. cambogia*/day	Significant body weight lost	[69]

**Table 4:** Summary of clinical studies of *Garcinia*/HCA that have shown none significant antiobesity effect.

Duration	Subject	Treatment	Outcome	References
1 day	Normal, groups: placebo, HCA of total 10 cyclists	4300 g HCA	No significant changes in total fat and carbohydrate oxidation rates	[70]

2 weeks	Normal-moderately obese, group: placebo, HCA, and HCA + MCT of total 7 males and 14 females	500 mg HCA; 500 mg HCA + 3 g medium chain triacylglycerols (MCT)	No significant differences in satiety, daily energy intake and body weight loss within all groups **Comment: subjects were under negative energy balance conditions (eliminating the possibility of *de novo* lipogenesis and reservation of glycogen reserves had occurred; thus, the only possible remaining mechanism increased hepatic fatty acid oxidation)	[71]

2 weeks	Normal-moderately obese, groups: placebo, HCA, and HCA + MCT of total 11 male	500 mg HCA; 500 mg HCA + 3 g medium chain triacylglycerols (MCT)	No significant differences in body weight reduction, EE, appetite ratings, and substrates oxidation (protein, fat, and carbohydrate oxidation) within all groups **Comment: 2-week intervention is too short	[41]

2 weeks	Normal-moderately obese, groups: placebo, HCA of total 10 sedentary males, with or without moderately intense exercise	3 g (−)-HCA daily	No significant difference in RQ, EE, and blood parameters during rest nor during exercise	[42]

8 weeks	39 subjects	1500 mg *G. cambogia *+ 300 *μ*g chromium picolinate/day	No significant difference in control and placebo	[72]

10 weeks	Overweight, groups: treatment: 29; placebo: 29	2 g *G. cambogia* extract daily	No clinically significant differences in body composition, plasma lipid profiles, antioxidant enzyme activity, and plasma adipocytokines	[73]

12 week	Obese, treatment: 5 M, 61 F; placebo: 14 M, 55 F	*G. cambogia* extract: 3000 mg of (50% of HCA); Diet: 5020 kJ/day (1200 kcal/day)	No significant difference in body weight and fat mass loss	[40]

12 weeks	Obese, groups: treatment: 36 F, 11 M; placebo: 41 F, 10 M	Botanical extract (1000 mg HCA daily)	No difference between placebo and treatment group but significant change of the body composition improvement index, body free fat mass, weight, BMI, and some other anthropometric measurements in both treatment and placebo groups	[74]

12 weeks	Obese, groups: treatment: 25 F, 7 M; placebo: 21 F, 5 M.	2.4 g *G. cambogia *(52.4% HCA) + 1.5 g *A. konjac *(94.9% glucomannan)	No significantvariation in body weight/other anthropometric and calorimetric parameters but significant hypercholesterolemic	[75]

12 weeks	BMI 28, groups: placebo: 23; treatment: 21	1,667.3 mg of *G. cambogia* extract/day (1,000 mg HCA/day)	No significant effect	[76]

**Table 5:** Commercialized dietary supplements that contain *G. cambogia* extract/HCA.

Product name	Company	Concentration of HCA	Doses recommended (daily)	Formulation of supplement
Super CitriMax HCA-600-SXS	Inter health N.I.	60% (−)-HCA in its free form, 1.0% (−)-HCA in its lactone form	3 capsules, 3 times daily, 30–60 min before meal	3 capsules per serving: *G. cambogia* extract 1500 mg (providing 900 mg of HCA), calcium 150 mg, potassium 225 mg, 0.5% sodium, 0.1% magnesium, 0.03% iron, 0.05% total phytosterols, 0.3% total protein, 4.5% moisture, and 8.5% soluble dietary fiber

GarCitrin	Sabinsa Corporation	50% (−)-HCA	500 mg, 3 times daily	500 mg of GarCitrin (providing 250 mg of HCA) and 5% Garcinol

Sci-Fit Pro Cut		50% (−)-HCA	4 capsules, 2-3 times daily, 30–60 min before meal	4 capsules per serving: *G. cambogia* 1000 mg (providing 500 mg of HCA), green tea extract 500 mg (98% polyphenols) (45% epigallocatechin/EGCG), guarana extract 910 mg (22% caffeine), caffeine 100 mg, L-carnitine 100 mg, white willow bark extract 100 mg (standardized for 15% salicin), dandelion 100 mg, juniper berry extract 100 mg, buchu extract 100 mg, and chromium 200 mg

*G. cambogia* and Kola Nut 450 mg	TerraVita	—	1 capsule, 3 times daily, with meals	225 mg *G. cambogia* fruit, 225 mg kola nut

*G. cambogia *	ProThera, Inc.	50% (−)-HCA	1–6 capsules daily, before meals.	500 mg *G. cambogia* (providing min. 250 mg of HCA), vegetarian capsule (hydroxypropyl methylcellulose, water), cellulose, magnesium stearate, and silicon dioxide

*G. cambogia* Plus	Atrium Inc	50% (−)-HCA	2 capsules, 3 times daily	Chromium 166 *μ*g (as Cr picolinate 500 mg and Cr arginate 160 mg), *G. cambogia* 340 mg (providing 170 mg of HCA), atractylodes 80 mg, citrus aurantii 80 mg, gelatin, rice powder, and magnesium staerate

*G. cambogia* Plus	BioCare Ltd.	50% (−)-HCA	3 capsules daily, 20 min before food	*G. cambogia *500 mg, vitamin B5 (calcium pantothenate) 6 mg, vitamin C (ascorbic acid) 5 mg, manganese gluconate(providing 270 *μ*g elemental manganese) 2.5 mg, and chromium polynicotinate 0.9 mg (providing 100 *μ*g elemental chromium)

*Garcinia* 1000	Source Naturals	50% (−)-HCA	1 tablet, twice daily, 1 hour before meal	Chromium (as chromiumpolynicotinate [ChromeMate] and chromium picolinate) 150 *μ*g, sodium 25 mg, *G. cambogia* fruit extract (providing 500 mg of HCA) 1 g

*Garcinia* 1000 hydroxycitric acid	Nature's life	50% (−)-HCA	—	*G. cambogia* rind concentrate (providing 500 mg of HCA) 1 g, cellulose, silicon dioxide, magnesium stearate and “*Micro-Cellulose*” coating

Citrin	Natural Nirvana	—	1 capsule, 2 times daily	*G. cambogia* 495 mg, BioPerine 5 mg

HCA 450 mg tablet	Higher nature	450 mg HCA per tablet	1-2 tablets, 3 times daily, 30 min before meal	Tamarind fruit extract, microcrystalline cellulose, magnesium stearate (vegetarian source), hydroxypropyl methylcellulose coating, silicon dioxide, and acacia powder

HCA Hydroxy citric acid	Viridian Nutrition Ltd.	50% (−)-HCA	1 capsule, 3 times daily, before meal	*G. cambogia* (providing 250 mg HCA) 500 mg, viridian bilberry extract, alfalfa, spirulina blend 150 mg, and vegetarian cellulose capsule 120 mg

HCA hydroxycitric acid	Life Extension Foundation	50% (−)-HCA	1 capsule, 3 times daily, 45 min before meals with 1 capsule of CitriChrome	*G. cambogia* (fruit) (providing 250 mg of HCA) 500 mg

Hydroxycitrate	Solgar	50% (−)-HCA	1 capsule, 30–60 min before meal	*G. cambogia* fruit powdered extract (providing 250 mg [50%] HCA) 500 mg, hydroxypropylmethyl cellulose, vegetable magnesium stearate, and silicon dioxide

Hydroxycitrate Plus	Metagenics	—	1 tablet, 3 times daily, 30–60 min before meal	*Garcinia* fruit extract (*G. cambogia*) 500 mg, L-carnitine 100 mg, niacin (as niacinamide) 50 mg, pantothenic acid (as D-calcium pantothenate) 25 mg, riboflavin 10 mg, manganese (as manganese arginine) 750 *μ*g, and chromium (as chromium nicotinate glycinate) 75 *μ*g
